# Usability study of a simplified electroencephalograph as a health-care system

**DOI:** 10.1186/s13755-015-0012-z

**Published:** 2015-11-17

**Authors:** Shinichi Motomura, Muneaki Ohshima, Ning Zhong

**Affiliations:** Maebashi Institute of Technology, Kamisadori 460-1, Maebashi, Japan; Ikuei Junior College, Takasaki, Japan

**Keywords:** EEG, Simplified electroencephalograph, Mobile EEG device, Healthcare

## Abstract

A healthy lifestyle is becoming increasingly important worldwide, and various health monitoring devices that support this trend are currently being developed. Devices measuring blood pressure, weight, temperature, and pulse have been mainstream. In contrast, electroencephalography has been only useful in medical practice and brain research. For an electroencephalograph to be used in health care, it must be small and user-friendly. The conventional electroencephalograph uses more than twelve electrodes attached to a user’s head with paste and hence is very precise. In contrast, the simplified electroencephalograph has inferior precision, but it is cheap, lightweight, paste-free, and requires only a short setup time. In this study, we verified the usefulness of the simplified electroencephalograph in investigating the mental condition of persons. We present interesting results associated with the setting position of the electrodes, the behavior of brain waves during work, and the appearance of individual differences. Consequently, we predict that this simplified device will be widely used for health diagnosis.

## Background

People are becoming increasingly concerned about their health, and it is believed that monitoring living condition and health status is essential for longevity. Pervasive utilization of health monitoring systems began in the 1980s, and currently, blood pressure and heart rate can be recorded at home. Since the year 2000, significantly advanced wearable devices have entered our daily lives. Miniaturization of the electroencephalograph is also progressing. Thus, in the near future, a new aspect of mobile health will be monitoring brain waves at home. This will help simplify disease diagnosis via telemedicine. In this study, we verify the practicability of the simplified electroencephalograph in investigating human mental condition and consequently predict that this simplified device will be widely used for health diagnosis.

The simplified electroencephalograph has already been put to practical use, and the development of other electroencephalographic applications is also ongoing. Let us take the toys developed in Japan that are equipped with simplified electroencephalographs as an example. The first one is a cat ears-shaped toy. When the attention of the person wearing the ears is focused, the ears become erect. Conversely, they collapse when a relaxed state is detected. The second example is an application for affinity diagnosis in which the users of the application direct a simplified electroencephalograph stare at each other. The application can be used to determine whether the partner has goodwill or not based on electroencephalographic data. Another application that can manipulate Google Glass based on brain waves was developed in the UK. In this application, when the user focuses consciousness on the subject in front of him/her, a picture is taken. The image can then be shared. Thus, the simplified electroencephalograph is not only for toys. It is expected to become a next-generation communication tool and input device.

To date, the electroencephalograph was only helpful in developments in the brain science and medical fields. The conventional electroencephalograph utilizes more than twelve electrodes that are attached to the head with paste. In addition, measurements are taken an electrically isolated room called a shield room in which extremely high-precision readings can be obtained. Thus, it has good precision, but requires a long period of preparation. In contrast, the simplified electroencephalograph has inferior precision, but is cheap, lightweight, paste-free, and the setup time is short. Moreover, it is equipped with a rechargeable battery, so an external power supply is unnecessary. However, spatial information about the electric potential cannot be obtained because the number of electrodes it possesses is too small. The battery gives approximately 1–2 h of consecutive measurements.

Thus, the simplified electroencephalograph has multiple limitations. Consequently, there are many challenges that must be overcome before it can be used for a more practical purpose such as health care. The first is the problem of placement of the electrodes. In general, to facilitate easy setup, only a few electrodes are used in the central part of the head. However, depending on the purpose of the measurements, there is a possibility that the left side and the right side are more advantageous than the central part. The second is the problem of capturing changes in physical conditions in everyday life using a simplified electroencephalograph. In everyday life, a person’s condition varies from favorable, to poor physical condition, to fatigue. Of course we can ascertain the state from electroencephalographic data if the data are from a conventional electroencephalograph that has many electrodes. Thus, it is necessary to investigate the differences in the simplified electroencephalograph and its many limitations. This study used B3 Band? a simplified electroencephalograph device developed by B-Bridge International. The results of our study show that this simplified electroencephalograph is practicable for health care.

The remainder of this paper is organized as follows. "Related work" discusses related research on health management using a simplified electroencephalograph. "Methods" outlines the methodology used. "Investigation of the practicability of the simplified electroencephalograph" reports on the results of a stress experiment conducted in which brain wave changes in daily life were investigated. "Discussion" discusses the feasibility of using a simplified electroencephalograph for a health-care system. Finally, "Conclusion" concludes this paper.

## Related work

Studies on the practical and effective use of simplified electroencephalographs are currently being conducted. In addition, research is also being conducted on health management applications, of which studies that explore the relationship of EEG and mood are just one example. Yoshida et al. used a simplified electroencephalograph to conduct analysis of brain waves during the learning state [1–3]. In the present study on the degree of stress and concentration, the ratio of $\beta$ to $\alpha$ waves that Yoshida et al. used as indicators has proven to be effective. Ishino et al. used a simplified electroencephalograph to conduct a study on emotions and sensations [4]. However, the simplified electroencephalograph is rarely used in the full-fledged medical and brain science fields; consequently, the number of such case studies is low. Chang et al. conducted a study of a system that alerts a person of his/her health state [5]. They developed a system that informs a physician when the person is in a dangerous state based on statistics from the medical field. Diego et al. verified the EEG effects of aromatherapy [6]. They primarily focused on alpha and beta waves, and showed the effectiveness of the aromatherapy.

## Methods

This study used a percentage of the frequency components acquired by the B3 Band a simplified electroencephalograph device developed by B-Bridge International. On that basis, we tried to answer the following three questions: (1) When the position of the electrode of the simplified electroencephalograph changes, is the frequency component the same or different? (2) Are variations in EEG operating consecutively caught precisely? (3) Does it detect individual differences? We performed two experiments related to the position of the electrodes and consecutive work. The brain waves collected were then analyzed in terms of the ratio of a frequency component, not a wave pattern. This is to determine which band is dominant. In the experiment conducted to find the most suitable electrode position, we tried to ascertain the electrode position at which differences between calculation activity and relaxation gives the greatest signal. Therefore, we examined the significant differences and discussed in terms of distance and a coefficient of correlation. In the experiment on consecutive work, we investigated how the frequency ingredient ratio changes based on questionnaires given to subjects. For the question of individual differences, we compared how the frequency component ratio differs between persons mentally using a virtual abacus and general calculation activities. We consider whether a feature can be found in the brain waves of the simplified electroencephalograph when the load expected in everyday life is applied. Consequently, we show that a simplified electroencephalograph is useful for health care.

## Investigation of the practicability of the simplified electroencephalograph

**Fig. 1:**
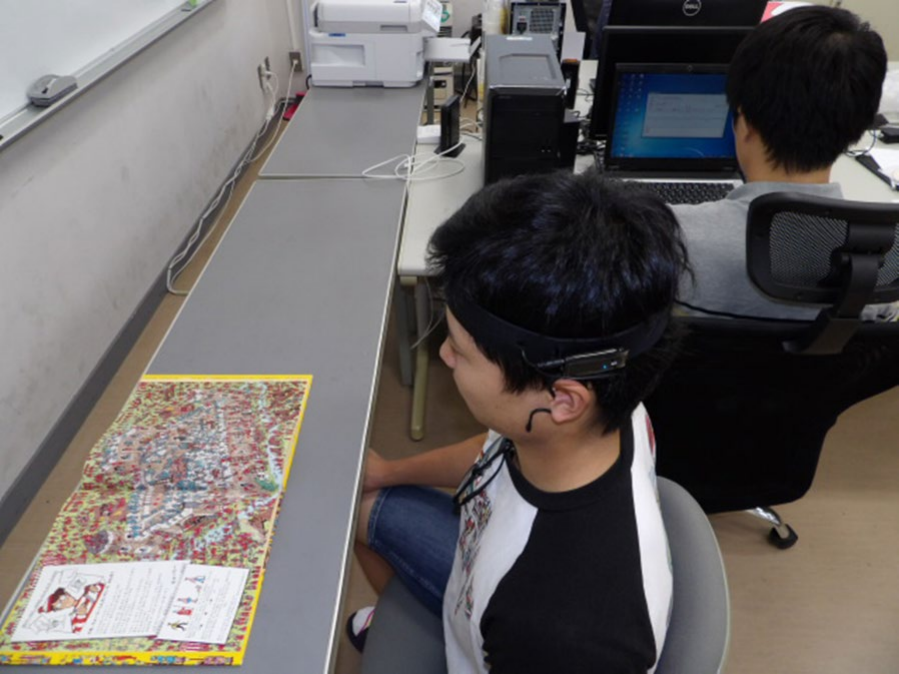
Room situation. The state of the operator and the subject

**Fig. 2:**
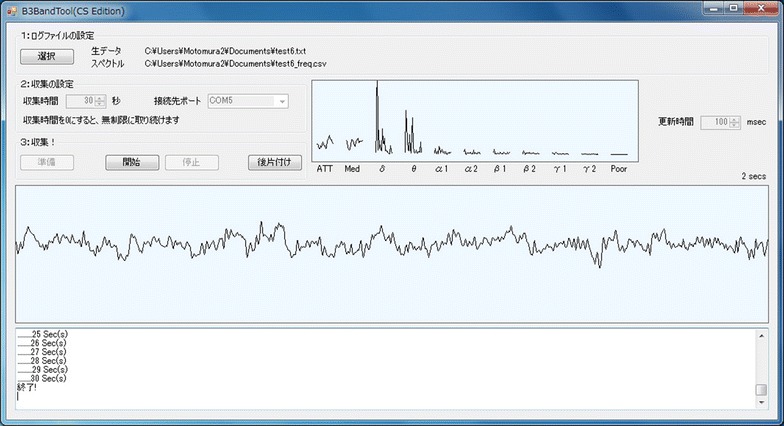
Experimental tool. The EEG observation and recording tool (original tool)

In this study, we investigated whether a simplified electroencephalograph is practicable for health care. We determined the characteristics of the electroencephalograph in this study and examined the position of the electrode in contact with the forehead. In addition, we collected electroencephalographic data during a period of fatigue assumed to be experienced in everyday life. In this way, we investigated the practicability of the simplified electroencephalograph. We conducted the experiment in the room shown in Fig. 1. The subject on the left donned a simplified electroencephalograph and performed an experimental task. The operator on the right explained the task and recorded the brain waves via a PC. To conduct the electroencephalographic observation and recording we used a tool that we developed, depicted in Fig. 2. With this tool, brain waves were displayed on the central part of the screen in real time, and the frequency component was displayed in the top right corner. In addition, log data were saved to an operation PC.

### Characteristics of the simplified electroencephalograph

**Fig. 3:**
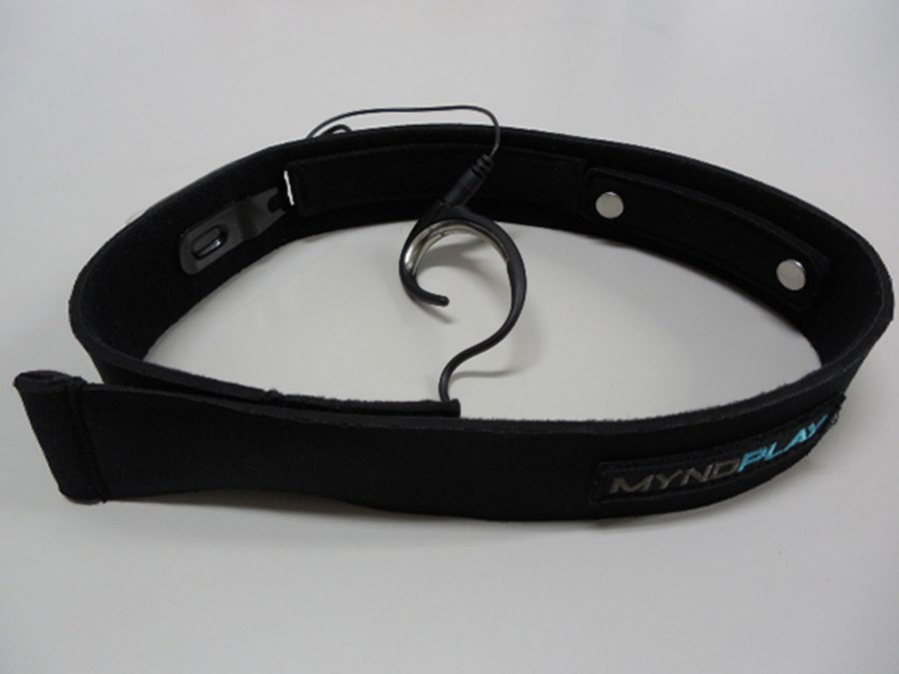
B3 Band. The simplified electroencephalograph devices developed by B-Bridge International

The simplified electroencephalograph used in this study was a B3 Band (depicted in Fig. 3), a headband type device produced by the B-Bridge Company. The B3 Band uses NeuroSky chips, and is well-known worldwide [7, 8]. A metal moiety in the form of an ear is connected to the B3 Band and a cable connects it to an earlobe, with standard electric potential. The potential difference is measured via two electrodes buried in a part of the band. However, the number of effective channels is one bipolar derivation. Bluetooth is utilized to connect the simplified electroencephalograph to a computer; thus, it is not restricted by a cable. Its weight is approximately 100 g, and even long-term measurement is not tiring, but sufficient care is necessary to ensure that the belt is not overly tight. In addition, according to the shape of the earlobe and sweat status, measurement may prove problematic.

For data acquisition, the raw data (low data) are measured at a sampling frequency of 512 Hz. In addition, the frequency spectrum data of the intensive and relaxation degrees are measured once per second. The data acquired by B3 Band are sorted according to frequency band, as shown in Table 1.

**Table 1:** Frequency band division of B3 Band

Band name	Apparatus output name	Frequency band (Hz)
Delta	Delta	0.5–2.75
Theta	Theta	3.5–6.75
Low-alpha	Alpha1	7.5–9.25
High-alpha	Alpha2	10–11.75
Low-beta	Beta1	13–16.75
High-beta	Beta2	18–29.75
Low-gamma	Gamma1	31–39.75
High-gamma	Gamma2	41–49.75

### Study of optimal electrode position

In conventional measurement of brain waves, electrodes are attached to the scalp based on the international 10–20 system or extended international 10–20 system. On the other hand, the simplified electroencephalograph uses an electrode placed on the upper part of the central forehead. However, it is unclear whether the location of this electrode is the most suitable for examining the differences in brain activity. Consequently, we proposed four patterns for setting the location of the electrodes, including whether to place the ground electrode on the right or left ear, and whether to place the signal input electrode in the center of the forehead or not. When the electrodes for measuring brain waves are based on the extended international 10–20 system, we can use positions F9, Fp1, Fpz, Fp2, and F10 [9, 10]. In addition, we can use the left ear (A1) and the right ear (A2) as reference points. We investigated these combinations of candidate positions.

#### Experimental methodology

**Fig. 4:**
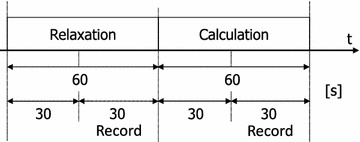
Timing chart 1. The timing chart of the arithmetic experiment

Table 2 shows data for 20 healthy subjects from 18 to 35 years old (19 males, one female) from whom measurements were obtained in the four electrode positions. There were two tasks: closed-eyes in a relaxed state and closed-eyes while doing a mental arithmetic task. The closed-eyes mental arithmetic task entailed subtracting seven continuously from the same initial value. The initial value consisted of the four numbers 230, 250, 270, and 290, with the presentation order of the initial value for each subject changed in the experiment. Measurement time for each task was 1 min, and data from the last 30 s were used. Figure 4 shows the timing chart of the experiment.

**Table 2:** Combination of electrode position and tasks

	Measuring electrode	Earth
(a)	F9·Fpz	A1
(b)	Fp1·Fp2	A1
(c)	Fp1·Fp2	A2
(d)	F10·Fpz	A2

After the measurement, the frequency spectrum data of the second 30 s were extracted. Then, the data were represented by component ratio as a percentage for each band in order to eliminate differences in power caused by individual differences. On this basis, group analysis was applied for the percentage of data aggregating the individual results of the 20 subjects. Combinations of certain electrodes were also evaluated to determine the difference between the percentage of relaxation and mental arithmetic task.

#### Experimental results

**Fig. 5:**
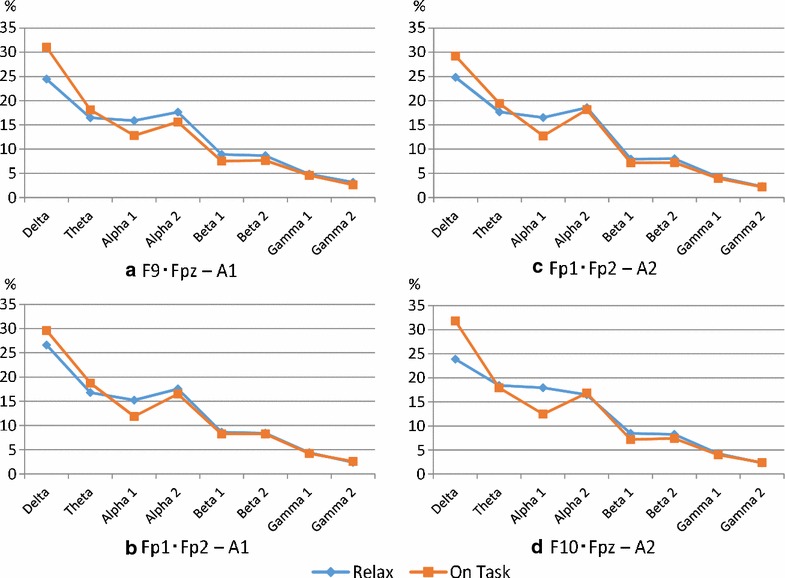
Experimental result. The frequency difference each electrode position

Figure 5 show the frequency band according to the ratio of the relaxed state and the mental arithmetic task at each electrode position. The horizontal axis represents the frequency band, and the vertical axis indicates the percentage. For example, we can consider the ratio of delta wave at relaxation to be 24.5 % in Fig. 5a. The frequency component was standardized, a sum of 100 and an average of 12.5 were acquired.

The overall trend is that the proportion of the delta and theta band is higher during the calculating activity task than in the relaxed state. On the other hand, in the alpha 1 band, the task percentage of On-task was reduced. There is virtually no difference between the bandwidth of the beta and gamma of the high-frequency band and that of the alpha 2 band.

In order to verify whether differences exist in each task, we first calculated the variance and performed an F test with a significance level of five percent. No significant difference was observed in any electrode location. However, these two tasks should indicate different distributions because different tasks were being processed. Therefore, we calculated both correlation coefficient and the distance between rows of data, and examined the electrode location that showed the largest distance and lowest correlation coefficient. Squared Euclidean distance was adopted to simplify comparison of the values.

Table 3 shows the variances for each task. The F statistics, P value, correlation coefficients, and distances of each electrode position are also presented. We focused on the difference between relaxation and calculation, and discussed which electrode position exhibited a major difference. Table 3 shows that the difference was greatest when the position of the reference point was A2 and the measurement electrodes were F10–Fpz. The second is the case of the reference point being A1 and the measurement electrodes F9–Fpz. On the other hand, when a measurement electrode is located in the center, Fp1–Fp2, of the forehead, the difference is virtually the same regardless of the left and right reference parts.

**Table 3:** The difference between relax and mental arithmetic task

	Variance		F test	Correlation analysis				Euclid distance
	Relax	Ontask	F statistic	Full band		Alpha1 below		
				Coefficient	P value	Coefficient	P value	
(a)	46.39	74.21	0.55	0.96	0.0001	0.97	0.1678	7.94
(b)	56.33	68.89	0.80	0.98	0.0000	0.96	0.1699	5.05
(c)	55.72	74.24	0.71	0.97	0.0000	0.96	0.1817	6.18
(d)	52.12	81.46	0.57	0.93	0.0007	0.92	0.2557	9.82

### EEG collected during fatigue

In everyday life, the degree of physical condition and fatigue will change. When using a simplified EEG for health care, it is necessary to detect this difference. It is advantageous to use a conventional EEG with many electrodes for this also; however, we have to use a simplified electroencephalograph assuming home use. Therefore, focusing on human fatigue, this study verified discovery of the difference using only one electrode.

#### Experimental methodology

**Fig. 6:**
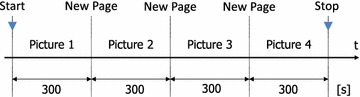
Timing chart 2. The timing chart of the Wally experiment

Five healthy male subjects ranging in age from 18 to 35 years old worked continuously for 20 min playing the world-famous game “Where’s Wally?”. The subjects did not need to turn the page as experiment auxiliary personnel turned the page every 5 min. Figure 6 shows the timing chart of the experiment. Measurements were performed in the relaxed and open eyes state, with placement of the electrode at the small F9-Fpz-A1, where the difference is lowest. The open eyes state EEG during the relaxed state was measured prior to the start of the experiment. Further, the frequency component data were recorded halfway through the experiment. In addition, in order to understand the situation after the experiment, it was terminated after 1 min following the end of the problems.

#### Experimental results

**Fig. 7:**
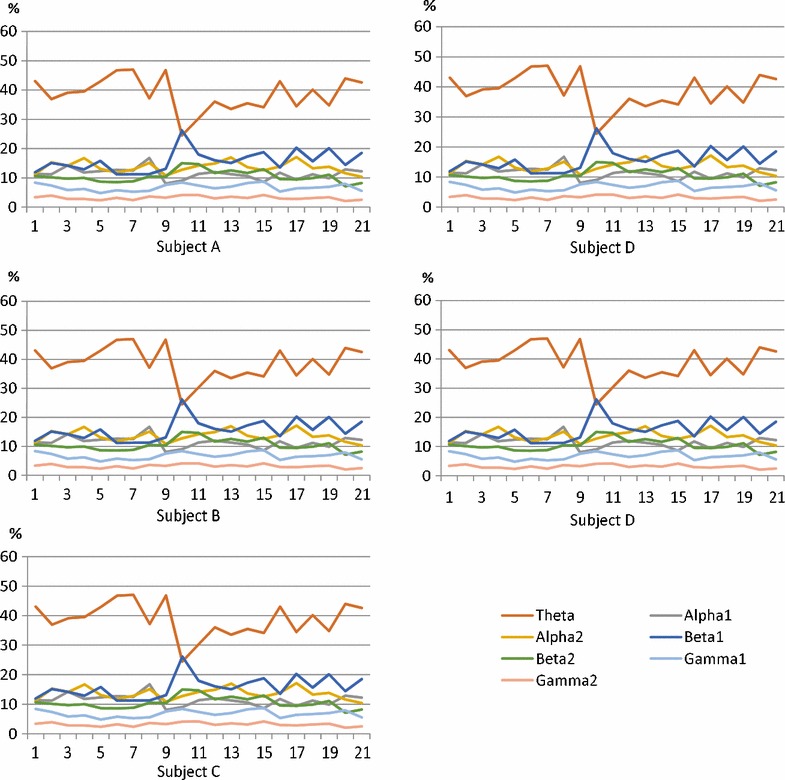
Fluctuations. Fluctuations in the frequency band by the ratio during continuous operation

Figure 7 show the variations in the frequency band by ratio during continuous operation. The horizontal axis represents time and the vertical axis shows the percentage. However, the time axis used to calculate the percentage is based on the integration of the power for one minute so that global variations were captured.

Figure 7 indicate the pattern of frequency variation for each difference. In particular, the theta band has the largest difference. The theta band patterns of the upper and lower variability all show different results. The alpha band was divided into two cases per subject. Increases in the second half case did not change the overall value. In the beta band, some subjects showed a variation in the middle and end, but there was no significant change. In the gamma band, there was no significant variation in any subjects.

In this experiment, the alpha band decreased during the experiment. In addition, increases in the beta and gamma band are shown. On the other hand, at 20 min of continuous operation, it was not possible to find a specific high-frequency feature in the beta band.

## Discussion

### Consideration of optimal electrode position

Table 3 shows the arrangement of the largest electrode for the difference between the frequency ratio data at the time of calculation activities and the relaxed state, the reference point A2, and the electrodes F10–Fpz. This analysis shows a correlation perspective except for the difference in percentages.

When using the ratio data for all bands, the combination of reference part A2 and electrodes F10–Fpz exhibit the most for the decision as well as the difference in percentages. Thus, this electrode position resulted in the biggest difference in distance and correlation. The big factor of this result is a ratio of alpha 1 wave. Comparison of calculation activity and relaxation showed that the ratio of alpha 1 waves decreases during calculation. For this decreased width, the electrode position exhibited the largest value.

### Study of continuous work during EEG data collection

In this experiment, we focused on the fact that the frequency band by the proportion of the variation pattern is different for each of the five subjects. Here, we summarize the results of the interview conducted with the subjects for concentration. Subject A said that he sometimes felt less focused. Subject B said that the second half was a little tiring. Subject C said he concentrated the most during the middle of the first half. Subject E experienced a refreshing feeling when the page was new. However, subject E said he gradually grew tired. The variation corresponds to the listening content effected to the theta band. It is clear that the ratio of the theta band decreases with poor concentration. This result indicates that it is possible to find the difference in concentration of the subject using a simplified electroencephalograph. On the other hand, instantaneous fluctuations for unrelated waveforms associated with blink and motion were measured several times during the experiment. This is expected to change when the characteristic issues of the subject are resolved. However, because the system for simultaneously measuring the discovered signs and EEG of subjects is not yet complete, this verification is an important consideration for the future.

On the other hand, we were not able to discover the EEG associated with fatigue in this experiment. We believe that this was because the subjects could not be sufficiently fatigued doing continuous work for only 20 minutes. Therefore, more time is necessary to consider physical fatigue and mental fatigue from the perspective of long-term observation.

### Wide inter-individual variability in a mental arithmetic task

**Fig. 8:**
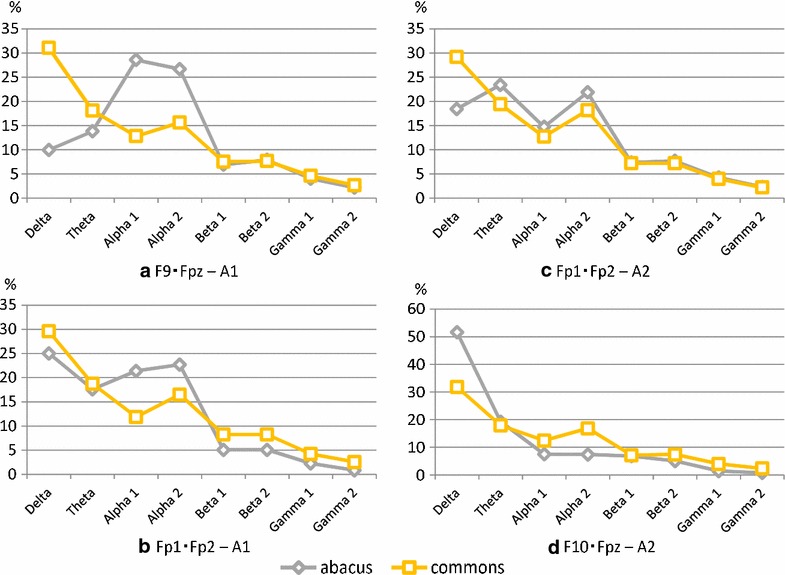
Individual difference. Wide interindividual variability in the task of mental arithmetic

In the experiment considering the differences between the relaxed state and that of the mental arithmetic task, we compared one person's mental abacus skills with that of 20 ordinary persons. Figure 8 show the differences in the frequency ratio of four electrode set patterns during the task of mental arithmetic.

Table 4 shows the variances for each task. The F statistics, P value, correlation coefficients, and distances of each electrode position are also presented, in the same manner as in Table 3. We focused on the difference between the abacus skill subject and the common subjects, and examined which electrode position exhibited the most difference. Firstly, in order to verify whether differences existed in each subject, we calculated the variance and performed F test with a significance level of five percent. However, no significant difference was observed in any electrode location.

**Table 4:** The difference between presence or absence of abacus skill

	Variance		F test	Correlation analysis				Euclid distance
	Abacus	Normal	F statistic	Full band		Alpha1 below		
				Coefficient	P value	Coefficient	P value	
(a)	87.38	74.21	0.83	0.35	0.3897	−0.85	0.3514	28.93
(b)	89.68	68.89	0.74	0.87	0.0053	0.51	0.6561	13.36
(c)	58.54	74.24	0.76	0.86	0.0056	0.23	0.8534	12.27
(d)	247.53	81.46	0.17	0.93	0.0009	0.97	0.1471	22.88

Table 4 shows that the difference was greatest when the position of the reference point was A1, and the measurement electrodes were F9–Fpz. The second is the case of the reference point being A2 and measurement electrodes F10–Fpz.

It is clear from Fig. 8 that the alpha band for the person with abacus skill is significantly different from others. The subject explained that he performed calculations using a virtual abacus in his head. Thus, a difference appeared in comparison with the common subject. The simplified electroencephalograph has many limitations. However, it can detect the differences in such a characteristic.

## Conclusion

In this study, we conducted experiments using the simplified electroencephalograph B3 Band to determine whether this simplified device is practicable for health care. We verified the efficacy of the device for this purpose by placing the small electrode in various positions on the human head. Furthermore, we showed that it is possible to capture the variations in EEG that occur during continuous working using this simplified electroencephalograph, and also to detect the difference in related characteristics.

The results indicate that larger differences can be detected when the electrode is placed to the left or right side rather than in the center right of the head. Further, even with a simplified electroencephalograph, it is possible to clearly capture the differences between frequency components in continuous operation. In addition, we showed that we could easily determine concentration periods by analyzing brain waves.

On the other hand, in order to seriously use a simplified EEG in health care, it is necessary to consider the load and fatigue that occur in everyday life. Therefore, this kind of full-scale, long-term life observation in EEG monitoring will be implemented in the next stage.
