# Erratum to: Usability study of a simplified electroencephalograph as a health-care system

**DOI:** 10.1186/s13755-016-0014-5

**Published:** 2016-02-16

**Authors:** Shinichi Motomura, Muneaki Ohshima, Ning Zhong

**Affiliations:** Maebashi Institute of Technology, Kamisadori 460-1, Maebashi, Japan; Ikuei Junior College, Takasaki, Japan

## Erratum to: Health Inf Sci Syst (2015) 3:4 DOI 10.1186/s13755-015-0012-z

After the publication of this work [1], we noticed an error whereby Figure 7 had five charts representing data from different subject areas which are identical. The corrected Fig. 7 is given below:

**Fig. 7:**
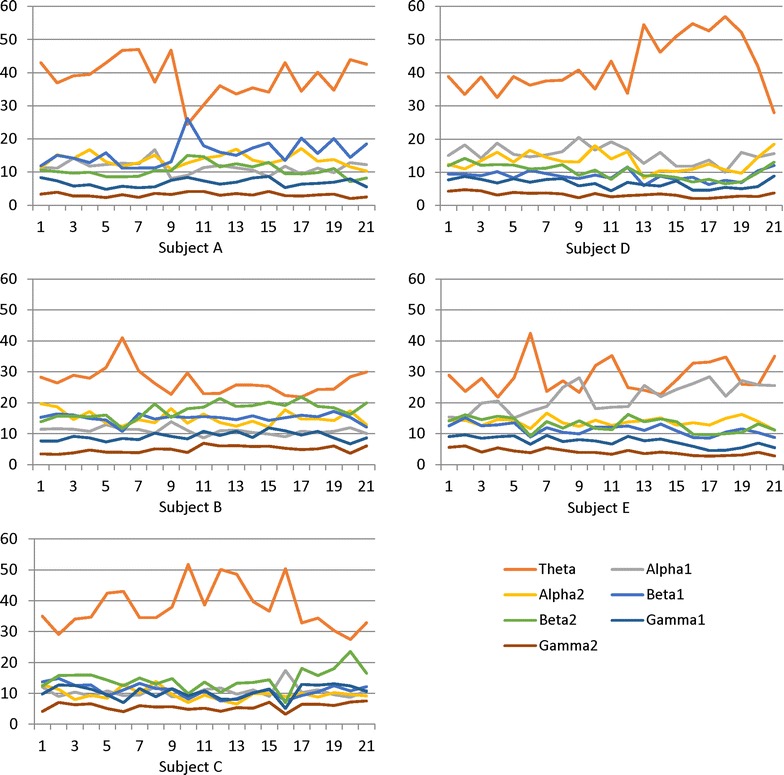
Fluctuations. Fluctuations in the frequency band by the ratio during continuous operation
